# Beyond the clicker: rethinking student response systems as pedagogical infrastructure for healthcare professions education

**DOI:** 10.3389/fmed.2026.1967058

**Published:** 2026-09-30

**Authors:** Rune Johan Krumsvik, Vegard Slettvoll

**Affiliations:** 1 University of Bergen, Faculty of Psychology, Bergen, Norway; 2 University of Bergen, Faculty of Medicine, Bergen, Norway

**Keywords:** artificial intelligence, feedback, formative assessment, healthcare professions education, large-group teaching, peer discussion, student response systems

## Abstract

Large-group lectures (150 students and upwards) remain a central component of healthcare professions education despite longstanding concerns about passivity, limited interaction, and difficulties in providing formative feedback at scale. These concerns have acquired renewed relevance as educators face digitally connected student cohorts, ubiquitous smartphones, and growing concerns about digital distraction. Student response systems (SRS), initially introduced through dedicated “clickers” and now commonly accessed through students’ own digital devices, offer one approach to transforming the large lecture from a predominantly transmission-oriented format into an interactive learning environment. Meta-analytic evidence indicates positive effects of SRS on cognitive and non-cognitive learning outcomes, while research across psychology, nursing, medicine, dentistry, and other higher education settings suggests that their educational value depends less on the technology itself than on the pedagogical processes it enables. In this *Perspective*, we argue for a shift from viewing SRS primarily as engagement technology towards understanding it as pedagogical infrastructure for retrieval, formative assessment, peer discussion, feedback, self-monitoring, and metacognitive calibration in large lectures. Drawing on nearly two decades of research and educational experience with response technology, we propose an evolution from *SRS 1.0*, centred on response and participation, through *SRS 2.0*, centred on formative feedback and dialogue, towards *SRS 3.0*, where artificial intelligence may enable adaptive questioning, misconception detection, personalised feedback, and instructor decision support. This development creates new opportunities for healthcare professions education, but also requires renewed attention to pedagogy, digital competence, privacy, cognitive offloading, and the role of the educator.

## 1 Introduction: the large lecture in a new digital context

The large lecture is one of the most persistent yet contested formats in higher education. For decades, critics have associated it with lecturer monologue, limited student participation, asymmetric communication, and difficulties in knowing whether students actually understand what is being taught. These limitations are particularly apparent when 150–300 students, or more, are present simultaneously. Yet large-group teaching remains educationally and economically important in universities and is common in medicine and other healthcare professions programmes.

The contemporary debate adds another layer. Today's students enter lecture theatres carrying powerful networked computers in their pockets. Smartphones can provide immediate access to information, communication, artificial intelligence (AI), and educational resources like SRS, but the same devices can facilitate social media use, multitasking, and digital distraction. The resulting policy response can easily become binary: screens versus no screens, devices versus device-free teaching. We argue that this framing is incomplete. A more educationally relevant question is whether digital devices can be deliberately incorporated into pedagogical designs that make students cognitively active rather than merely digitally occupied.

Student response systems (SRS), also termed audience response systems (ARS) or classroom response systems, provide an instructive case. Early systems relied on dedicated handheld “clickers”; contemporary systems commonly allow students to respond through smartphones, tablets, or laptops. The instructor can obtain an almost instantaneous overview of students’ responses and use this information to adjust instruction, initiate discussion, revisit difficult concepts, or provide feedback.

Our research programme on formative e-assessment in large lectures was initiated in 2007/2008 and has subsequently examined SRS and related response technologies across several educational contexts, including psychology, nursing, dentistry, and other areas of higher education (1–8). A central observation throughout this work has remained remarkably stable: the educational effect of response technology depends less on the device than on the pedagogical repertoire surrounding it.

This Perspective therefore addresses three guiding questions:

(1) What has two decades of research taught us about the effects of SRS on learning?
(2) Through which pedagogical mechanisms can SRS enhance large-group healthcare professions education?
(3) How should SRS evolve in an era characterised simultaneously by digital distraction and generative AI?

We argue that SRS should increasingly be conceptualised not as a classroom gadget, but as a form of pedagogical infrastructure capable of supporting formative assessment, retrieval, peer dialogue, feedback, and metacognitive calibration at scale.

## 2 What have we learned from two decades of SRS research?

The evidence base for response technology has developed considerably. Early research often concentrated on whether students liked clickers, whether they increased participation, and whether they made lectures more engaging. More recent research has increasingly examined cognitive learning outcomes and the mechanisms through which SRS-supported activities may influence learning.

Meta-analytic findings generally support positive effects. Hunsu et al. compared classrooms using and not using ARS and found small but significant positive effects on cognitive and non-cognitive outcomes, with effects moderated by factors including knowledge domain, class size, and the design of clicker questions (9). Castillo-Manzano et al. similarly concluded that ARS had a positive, although moderated, effect on examination performance (10). Chien et al. found that the benefits of clicker-integrated instruction extended beyond novelty effects and simple memorisation, and particularly emphasised the value of asking students to explain and justify their responses (11).

More recently, Akbay et al. synthesised 142 independent effect sizes from 68 studies. SRS use had a statistically significant small effect on cognitive learning outcomes (Hedges’ *g* = 0.47; *k* = 109) and a medium effect on non-cognitive outcomes (*g* = 0.66; *k* = 33) (12). Moderator analyses indicated that effects varied according to educational level, discipline, and type of response system. These findings are important because they move the discussion beyond the simple question of whether response technology “works”: effects are heterogeneous and depend upon context and implementation.

For healthcare professions education, the earlier BEME systematic review by Nelson et al. remains particularly relevant (13). Among 21 included comparative studies, 20 evaluated ARS in lectures and 14 involved undergraduate education. Fourteen studies reported significantly improved knowledge scores with ARS. However, larger differences tended to occur in non-randomised studies and when ARS-based teaching was compared with non-interactive teaching. This qualification is theoretically important. If SRS is compared with a conventional monological lecture, the estimated effect may reflect not only technology, but the transition from passive to interactive pedagogy.

This distinction underpins our perspective: technology should not be confused with the pedagogical mechanism through which learning occurs.

## 3 The technology is not the pedagogy

Our early work conceptualised SRS within formative e-assessment rather than primarily as educational technology (1, 2). One important concept was the *moment of contingency*: an instructional moment in which evidence about students’ understanding becomes available and teaching can be adapted accordingly. In a conventional lecture, lecturers may infer understanding from facial expressions, questions from a small minority of students, or their own assumptions. SRS can instead provide information from a much larger proportion of the cohort. This creates a simple but potentially consequential feedback loop (see Figure 1).

**Figure 1:**
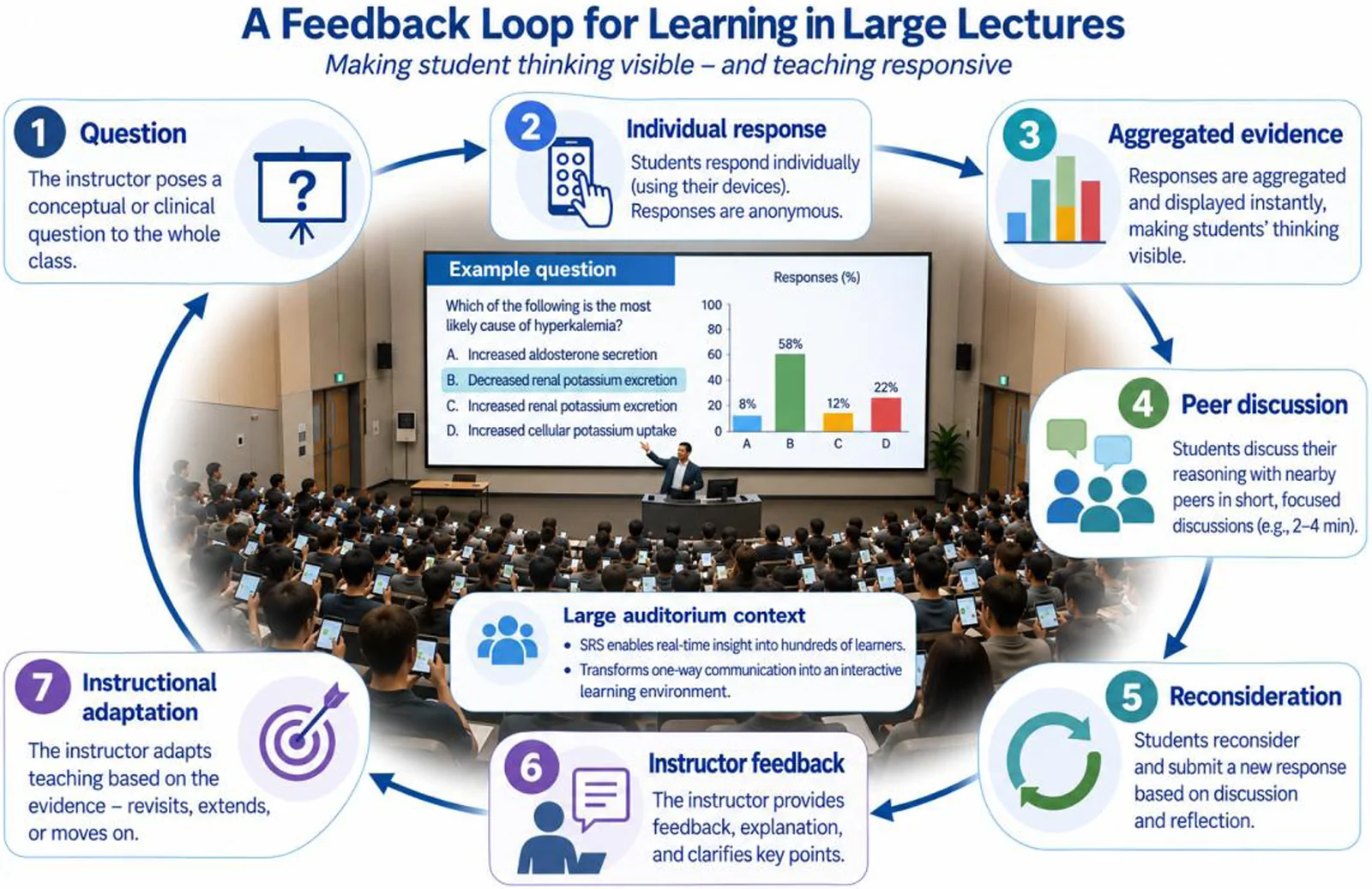
A feedback loop for learning in large lectures.

Research from our programme illustrates several components of this process. In large psychology lectures involving approximately 150–200 students, Ludvigsen et al. found that clicker questions created spaces for reflection and self-assessment and helped students monitor their own learning (3). Students who reported using clickers to monitor their understanding were also more likely to report using feedback in subsequent coursework, although an important “feedback gap” remained between receiving feedback and acting upon it.

Egelandsdal and Krumsvik subsequently showed how clicker interventions could support formative feedback in university lectures (4). In another study, brief peer discussions combined with response technology produced substantial changes in students’ responses (5). A larger mixed-methods study across 14 university lectures and 6,772 student responses similarly found that students perceived SRS feedback as supporting self-monitoring and understanding, while peer discussions improved performance (6). Importantly, lecturers also became more aware of students’ understanding.

This suggests that the educational sequence matters. A response submitted by a student is not, in itself, learning. The pedagogical potential emerges from what happens before and after the response.

Peer discussion appears particularly important. In later analyses of discussions occurring “behind the scenes” during SRS-supported activities, students engaged in explanation, argumentation, and critical reflection (7). Such processes can reveal something a histogram of correct and incorrect answers cannot: *why* students believe an answer is correct and how robust their conceptual understanding is.

Wood and Shirazi's systematic review of the student experience with ARS identified six interconnected themes: engagement, interaction, anonymity, questioning, instant feedback, and technological benefits and limitations (14). These themes correspond closely with observations across our own research programme.

The implication is that SRS should be viewed as an orchestration technology. Its function is not simply to collect votes. It can help lecturers orchestrate transitions between individual retrieval, collective visualisation of understanding, peer reasoning, lecturer explanation, and renewed assessment.

## 4 Why this matters for healthcare professions education

These mechanisms may have particular relevance for healthcare professions education. Medicine, nursing, dentistry, psychology, and related professions require students not only to acquire large bodies of knowledge but also to retrieve, integrate, and apply knowledge under conditions of uncertainty.

SRS in large lectures can therefore move beyond factual multiple-choice questions. Consider a clinical vignette and authentic video cases, followed by several plausible diagnostic or treatment options. Students can first commit individually to a response, indicate their confidence, discuss their reasoning with peers, respond again, and subsequently receive an expert explanation. Such a sequence potentially combines retrieval practice, formative assessment, peer learning, feedback, and metacognitive calibration.

This is important because professional competence requires students to recognise not only *what they know*, but also *what they do not know*. The difference between confidence and correctness can itself become pedagogically valuable information.

Evidence from nursing education illustrates these possibilities. Bingen et al. investigated nursing students learning physiology within a flipped-classroom design incorporating SRS (8). Students described SRS as contributing to a welcoming learning environment, encouraging participation, facilitating collaboration, and motivating study before and after campus sessions. However, the study also demonstrated that question difficulty and timing mattered: poorly calibrated questions could create stress rather than productive challenge.

Experience from dental education similarly suggests that response technology can be integrated into professionally oriented teaching rather than treated as an isolated technological intervention (15). These examples reinforce a broader principle: the value of SRS in healthcare professions education is likely to increase as questions move closer to authentic professional reasoning.

The relevant outcome is therefore not merely whether students answer more questions correctly during a lecture. Future research should examine whether repeated SRS-supported cycles of prediction, confidence judgement, discussion, feedback, and reconsideration contribute to clinical reasoning, diagnostic calibration, knowledge retention, and professional judgement.

## 5 From SRS 1.0 to SRS 3.0

We propose that the development of response technology can be understood through three overlapping generations.

### 5.1 SRS 1.0: response and engagement

The first generation centred on clickers and electronic voting. Its principal affordances were participation, anonymity, rapid aggregation of responses, and increased interaction in otherwise lecturer-centred environments.

### 5. 2 SRS 2.0: formative feedback and dialogue

The second generation is pedagogically more consequential. Smartphones and web-based platforms make response systems more accessible, but the central development is not technological. SRS becomes embedded in cycles of retrieval, peer discussion, formative feedback, self-monitoring, and instructional adaptation. The objective moves from “making lectures interactive” towards making student thinking visible.

A recent Norwegian study provides an important reminder that this remains relevant even after decades of digitalisation. Egelandsdal and Færstad found that first-year students preferred on-campus lectures and favoured digital questions combined with short peer discussions over lectures without student activity (16). Similarly, a 2026 study of contemporary SRS use shows both their potential for peer learning and students’ ambivalence when response systems are perceived as mechanisms of control (17).

We now see the contours of a third generation.

### 5.3 SRS 3.0: Adaptive and AI-supported response environments

Generative AI and learning analytics potentially change what a response system can do. Instead of lecturers manually producing every question, AI-supported systems could generate alternative clinical cases, adapt question difficulty, identify recurrent misconceptions, generate explanations at different levels, and provide instructors with summaries of where conceptual problems occur. In healthcare professions education, a future SRS might therefore support a sequence such as shown in Figure 2:

**Figure 2:**
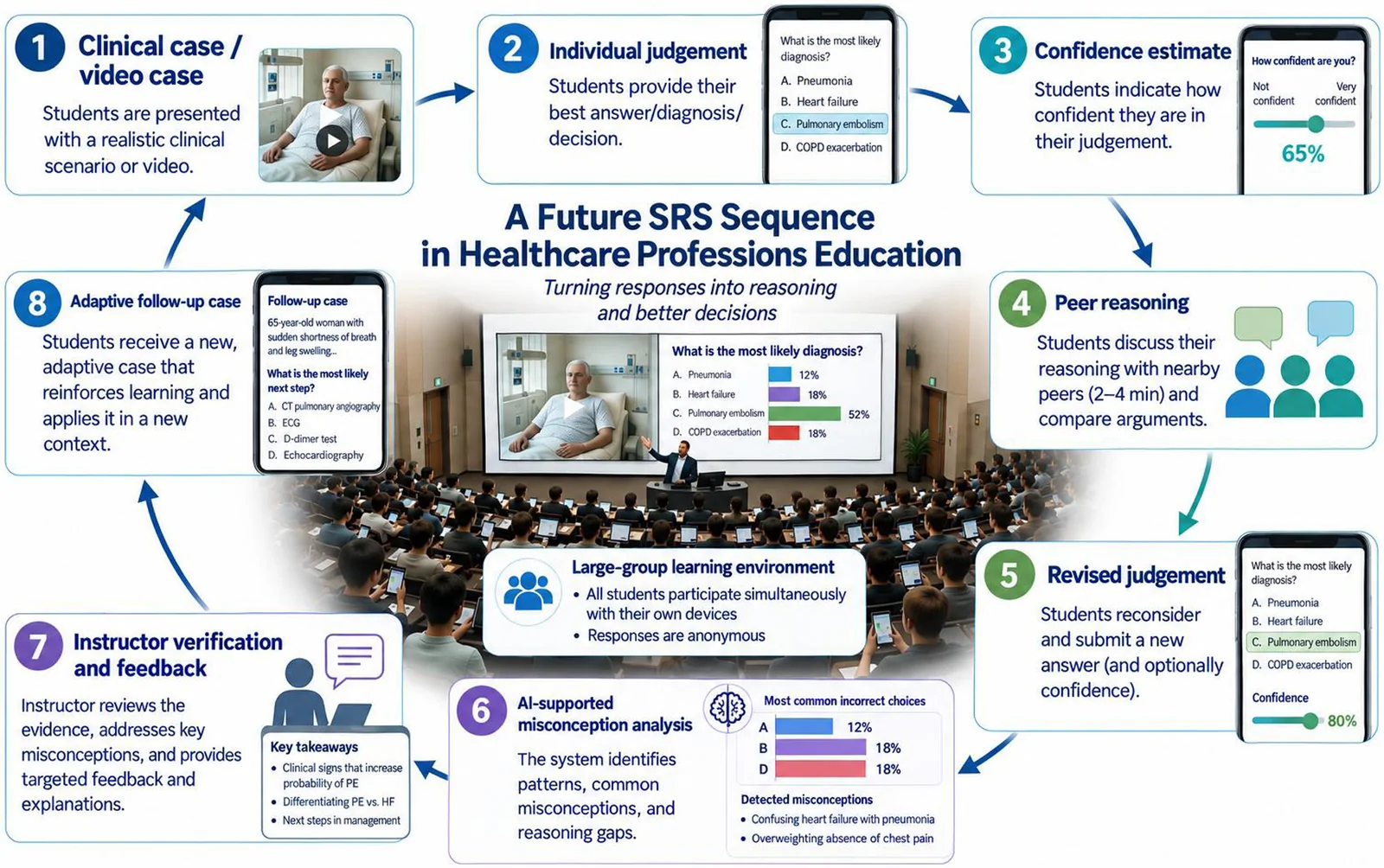
Turning response into reasoning and better decisions.

However, this transition should not be interpreted as an argument for automating the lecturer. The opposite may be more appropriate. As response systems become more powerful, the lecturer's pedagogical and professional judgement becomes increasingly important. AI-generated questions may contain errors, clinical reasoning can be context-sensitive, and personalised feedback may create an illusion of precision. Privacy, data governance, bias, explainability, and cognitive offloading also become increasingly important concerns.

SRS 3.0 therefore requires both digital competence and pedagogical-didactical competence from healthcare educators.

## 6 Discussion: From digital distraction to deliberate engagement

The debate surrounding smartphones in higher education is often framed around distraction. That concern is legitimate, but banning or ignoring digital devices addresses only one side of the problem. The same device that provides access to social media can also become an instrument for retrieval, feedback, reflection, and professional reasoning.

Nearly two decades of research on SRS suggest three conclusions.

First, the evidence indicates that well-founded use of student response systems can positively influence cognitive and non-cognitive learning outcomes, although effect sizes vary substantially across contexts (9–14). We should therefore move beyond asking whether SRS “works” and instead ask for whom, under which conditions, through which mechanisms, and for which outcomes.

Second, the pedagogical design surrounding the technology is crucial. Our own research has repeatedly highlighted formative feedback, self-monitoring, peer discussion, dialogical spaces, and moments of contingency as mechanisms through which SRS can contribute to learning (1–7). This distinction is especially important when interpreting intervention studies: comparing SRS-supported active teaching with a passive lecture risks attributing to technology what may primarily be an effect of improved pedagogy.

Third, healthcare professions education provides an especially promising context for the next generation of SRS. Clinical cases, video-cases, diagnostic uncertainty, confidence judgements, and professional decision-making offer opportunities to move response technology beyond factual recall. Rather than asking only “*What is the correct answer?”*, future SRS environments should increasingly ask students: “*How confident are you?”*, “*Why?”*, “*What evidence would change your decision?”*, and “*What did you misunderstand?”*.

Future research should therefore prioritise comparative studies that isolate pedagogical mechanisms, longitudinal studies of knowledge retention, studies of clinical reasoning and metacognitive calibration, and trials comparing conventional SRS with emerging AI-supported adaptive approaches. Studies should also investigate potential adverse effects, including distraction, superficial gamification, overreliance on automated feedback, and inequitable access to digital technologies.

The fundamental lesson from the clicker era remains relevant in the age of generative AI: technology does not create good pedagogy by itself. Its educational value arises when it helps teachers and students make thinking visible, respond to evidence of learning, and create meaningful dialogue at scale.

The future of response technology in healthcare professions education may therefore lie not in replacing the large lecture, but in transforming what can happen within it.

## Data Availability

The original contributions presented in the study are included in the article/Supplementary Material, further inquiries can be directed to the corresponding author/s.
